# A retrospective analysis of minimally invasive internal fixation versus nonoperative conservative management of pelvic ring fragility fractures and the elderly

**DOI:** 10.1186/s13018-023-03591-1

**Published:** 2023-02-15

**Authors:** Kaiwen Yang, Feifan Xiang, Junwu Ye, Yunkang Yang

**Affiliations:** 1 Department of Orthopaedics, The Affiliated Hospital of Southwest Medical University, No 25 Tai Ping Street, Jiang Yang District, Luzhou, 646000 Sichuan Province People’s Republic of China; 2 Sichuan Provincial Laboratory of Orthopaedic Engineering, Luzhou, Sichuan People’s Republic of China; 3 Department of Orthopaedics, Neijiang Hospital of Traditional Chinese Medicine, Neijiang, 641000 People’s Republic of China

**Keywords:** Fragility fractures of the pelvis, Treatment patterns for hip fractures, Minimally invasive surgical treatment

## Abstract

**Objective:**

We aimed to investigate the adoption of treatment patterns for hip fractures combined with minimally invasive surgical treatment of fragility fractures of the pelvis in older individuals and reviewed and analysed the treatment efficacy and feasibility.

**Methods and data:**

From September 2017 to February 2021, 135 older individuals with fragility fractures of the pelvis were admitted to our hospital. We retrospectively analysed patients who received surgical or conservative treatments. The general preoperative data, including sex, age, disease duration, cause of injury, AO/OTA type, BMI, bone mineral density, time from injury to admission, time from injury to surgery, ASA classification, number of underlying diseases, mean bed rest, clinical fracture healing, VAS score and Majeed functional score, were recorded.

**Results:**

The mean follow-up time for all 135 patients was 10.5 ± 3.6 months. Among 135 patients, 95 survived, and 11 and 29 patients passed after the surgical (mortality rate = 17.74%) and conservative (mortality rate = 39.73%) treatments, respectively. The average follow-up time for the 95 surviving patients was 14.5 ± 1.8 months. The Majeed and VAS scores for the operation group were significantly better than those of the conservative group. The bed rest and fracture healing times were also shorter in the surgical treatment group than in the conservative group.

**Conclusion:**

The use of a minimally invasive surgical treatment combined with the geriatric hip fracture treatment model to treat fragility fractures of the pelvis improved the quality of life in older patients.

## Introduction

Recently, incidences of hip fractures in the older population have increased annually [1, 2]. Concordantly, the number of older patients with fragility fractures of the pelvis (FFP) owing to low-energy injuries has also increased [3]. Previously, FFP in older individuals were treated more conservatively, considering the risks of anaesthesia and surgery. There are difficulties in reduction and fixation and a high risk of internal fixation failure in patients with osteoporosis [4, 5]. In contrast, after years of work by national and international experts, early surgery has been recommended for hip fractures in older individuals, regardless of the stability of the fracture type. The notion of “surgery within 48 h” has become widely accepted; therefore, the Israeli Ministry of Health has included it as a condition of payment for national health insurance, and the American Academy of Orthopaedic Surgeons has included it in its guidelines [6, 7].

FFP in older individuals occur in a similar location to geriatric hip fractures and increases the risk of osteoporosis, complex medical comorbidities, and anaesthesia. However, we lack an established management model for FFP in older individuals. The mortality rate of pelvic fractures is similar to that of hip fractures in older individuals [8]. Höch et al. [9] retrospectively compared surgical versus conservative treatment of 128 FFP in older individuals, estimating an overall mortality rate of 30% at the two-year follow-up with an all-cause mortality rate of 41% and 18% in the conservative and surgical treatment groups, respectively. These results suggest that surgical treatment may reduce bed-bound related complications and mortality.

The minimally invasive pelvic surgery techniques offer many advantages, such as less trauma, shorter operation time, and low risk of anaesthesia [10]. In the face of two similar fracture types, we can learn from successful cases of treating hip fractures to improve the quality of life and survival of senile pelvic fracture patients [11]. By establishing a fast-track system, we can conduct comprehensive preoperative assessments earlier. Reduction and fixation of fractures can promote early functional exercise, which will significantly improve the survival rate, quality of life and prevention and control of near and long-term complications [12]. Additionally, surgical guidelines for patients can be appropriately extended. The treatment of older individuals with poor tolerance for major surgery and additional complications is beneficial to patients as well as reduces the burden on their families and society. Therefore, treating geriatric pelvic fractures by combining the hip fracture treatment model with minimally invasive repositioning and fixation may improve the patient survival rate.

## Materials and methods

From September 2017 to February 2021, 135 older individuals with FFP were admitted to our hospital. We retrospectively analysed the patients who received surgical and conservative treatment and evaluated the efficacy and feasibility of minimally invasive repositioning and fixation of elderly FFP.

### Inclusion and exclusion criteria

We performed a retrospective study after obtaining approval from the Institutional Review Board. The inclusion criteria were as follows: (1) Age ≥ 65 years; (2) Pelvic fracture caused by low-energy injuries, such as falling from a standing position; (3) Treated with minimally invasive repositioning and fixation or conservative treatment; (4) Complete clinical data and (5) Follow-up time > one year. The exclusion criteria included (1) younger patients with a high-energy trauma; (2) pathological fractures, such as metastatic tumours, fractures caused by high doses of hormones and open fractures; (3) incomplete clinical and follow-up data; (4) those with less than one year of post-operative follow-up; (5) patients who died during the follow-up period; and (6) those with ASA classification IV or above [13]. Informed consent was obtained from all included patients.

### Observation items and methods

General information about the patients, including sex, age, duration of illness, cause of injury, AO/OTA classification [13], low body mass index (BMI) [15], bone mineral density, time to admission owing to injury, ASA classification and underlying disease, of the patients were recorded before surgery. The mean time in bed, clinical fracture healing, and VAS [16] and Majeed functional scores [17] were compared. Patients were discharged from the hospital for regular follow-up via general outpatient clinics or telephone, with post-operative follow-up at 1, 3, 6 and 12 months.

### Treatment modalities

We adopted the treatment modality for geriatric hip fractures, such as multiple organ function assessment, anaesthetic risk assessment, preoperative health education, pain control, anticoagulation, pulmonary function training and management of medical comorbidities, with two groups of patients.

#### Conservative treatment

The patient was immobilised in a special pelvic brace under the guidance of a rehabilitation physician and discharged to rest at home or transferred to a convalescent centre after stabilisation. Patients were also counselled regarding health and complication prevention and control.

#### Surgical treatment

Sacroiliac disruption/sacral fracture was addressed first using the standard iliosacral reduction techniques and percutaneous iliosacral screw osteosynthesis using cancellous screws. The internal fixator (INFIX) was used for an unstable pelvic anterior ring. Through a 3-cm vertical incision centred on the anterior inferior iliac spine (AIIS), the entry point on the AIIS was approached using meticulous dissection through the interval between sartorius and tensor fascia lata, taking utmost care to protect the lateral femoral cutaneous nerve [18]. The INFIX can also be placed outside the body. In the post-operative period, patients are provided support, such as respiratory support, non-steroidal analgesics, encouragement of deep breathing, anti-microbials (if necessary), and limb function exercises. Post-operative anti-osteoporosis treatment was provided according to a bone density assessment [19]. The INFIX can be removed 2–6 months after surgery, depending on the situation.

## Statistical processing

Continuous variables were expressed as mean ± standard deviation (SD) and were analysed using the Student’s *t *test. The chi-square test was used to analyse categorical variables. All statistical analyses were performed using SPSS 26.0 (SPSS, IBM, USA). Statistical significance was set at *P* value < 0.05.

## Results

A total of 135 geriatric pelvic fracture cases were selected from September 2017 to February 2021 in our hospital, which included 95 surviving patients. Among the 95 patients, 51 survived in the operation group and 44 in the conservative group. The surgical group consisted of 23 men and 28 women, with a mean age of 72.1 ± 5.6 years. AO/OTA classification revealed 18 cases belonging to type A, 28 cases of type B, and 5 cases of type C. The mean time to surgery owing to injury was 4.1 ± 2.4 days. The conservative group consisted of 19 men and 25 women, with a mean age of 73.4 ± 4.3 years. AO/OTA classification confirmed 11 cases in type A, 24 cases in type B, and 9 cases in type C. The mean time to admission owing to injury was 2.1 ± 2.4 days (Tables 1 and 2).

**Table 1:** Comparison of preoperative general data of two groups of patients with fragile pelvic ring fracture

	Cases	BMI (kg/m^2^)	Bone density (SD)	ASA classification				Underlying diseases				
				I	II	III	IV	Diabetes	High blood pressure	Lung infections	cerebral infarction	Others
Conservative Group	44	24.9 ± 4.2	− 2.7 ± 0.4	13	15	7	9	25	38	15	9	22
Surgical Team	51	26.4 ± 3.5	− 2.9 ± 0.6	18	21	10	2	27	35	13	5	29
Test value		*t* = − 1.899	*t* = 1.879	*x*^2^ = 6.309				*x*^2^ = 2.447				
*P* value		0.061	0.063	0.098				0.654

**Table2:** Comparison of preoperative general data of two groups of patients with fragile pelvic ring fracture

Group	Gender		Age (years)	Duration of illness (months)	Cause of Injury (cases)			AO/OTA typing		
	Male	Female			Falling down	Car accident	Other reasons	*A*	*B*	*C*
Conservative Group	19	25	73.4 ± 4.3	2.1 ± 0.6	21	13	10	11	24	9
Surgical Team	23	28	72.1 ± 5.6	1.7 ± 0.5	24	18	9	18	28	5
Test value	*x*^2^ = 0.035		*t* = 1.253	*t* = 3.544	*x*^2^ = 0.546			*x*^2^ = 2.639		
*P* value	0.851		0.213	0.001	0.761			0.267		

**Fig. 1:**
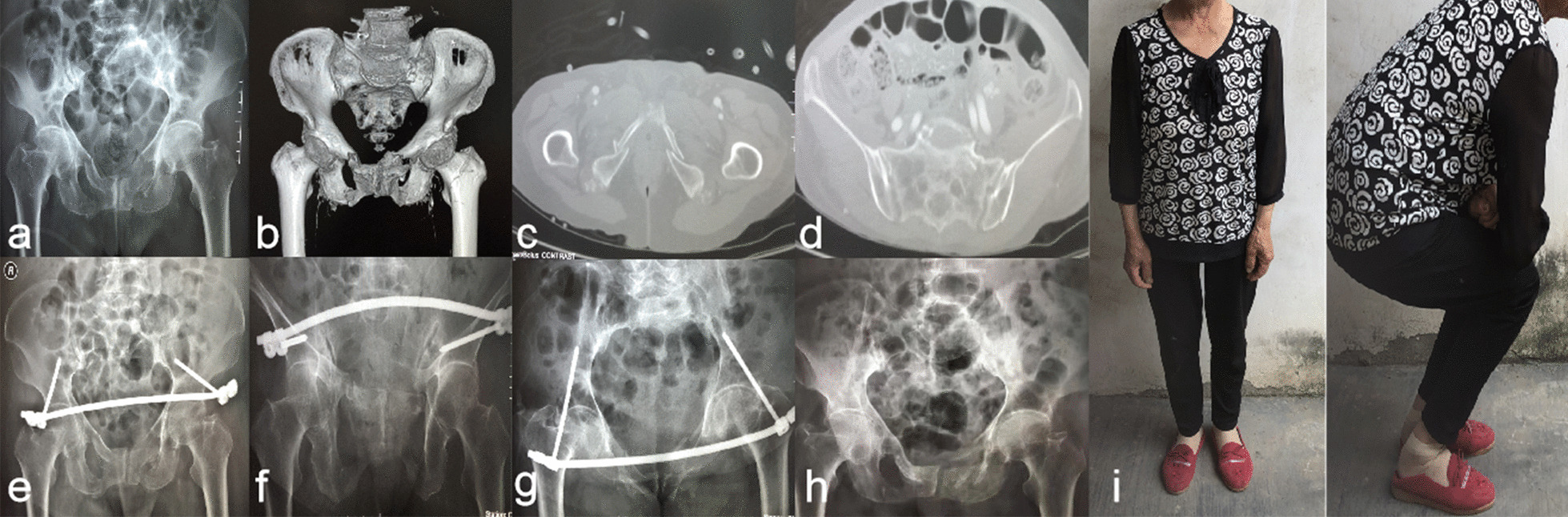
Typical case 1: A 75 years old female patient with pelvic ring fractures (AO/OTA type A) caused by a fall. The patient had a combination of gastrointestinal bleeding, moderate osteoporosis, anaemia, hypoproteinemia and diabetes mellitus. Treatment patterns of hip fractures were used pre- and post-operatively. The patient was treated with a minimally invasive closed reduction and internal fixation by the INFIX 5 days after injury. **a–d** Preoperative X-ray film and CT 3D reconstruction showed comminuted fractures of the upper and lower branches of the pubis. **e–g** The pelvic AP, outlet and inlet X rays. **h** Post-operative radiograph at 6 months after the INFIX stent removal, showing good fracture healing. **i** Post-operative functional images at 13 months

**Fig. 2:**
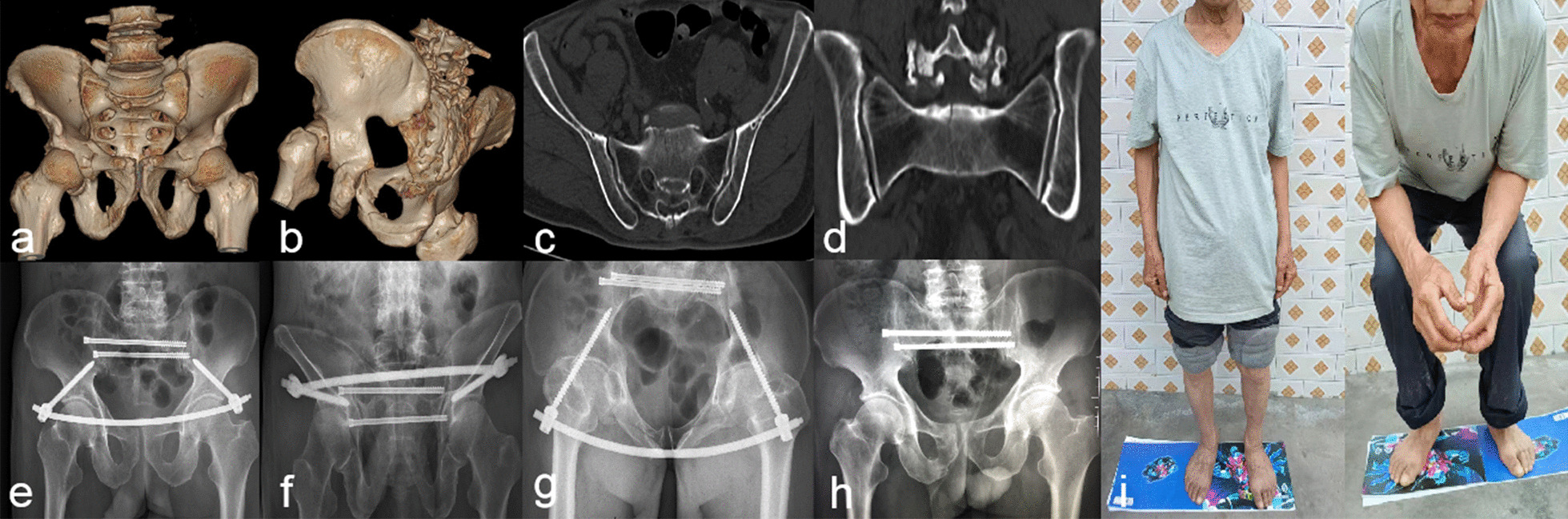
Typical case 2: A 82 years old male patient with pelvic ring fractures (AO/OTA type B) caused by a traffic accident. The patient had a combination of moderate osteoporosis, anaemia, hypoproteinemia, coronary heart disease and diabetes mellitus. Treatment patterns of hip fractures was used pre- and post-operatively. The patient was treated with a minimally invasive closed reduction and internal fixation by the INFIX 3 days after injury. **a–d** Preoperative X-ray film and CT 3D reconstruction showed comminuted fractures of the upper and lower branches of the pubis. **e–g** The pelvic AP, outlet and inlet X rays. **h** Post-operative radiograph at 5 months after the INFIX stent removal, showing good fracture healing. **i** Post-operative functional images at 12 months

All were followed up for a total of 10.5 ± 3.6 months. Among 135 patients, 95 survived, with 11 patients dying after surgical treatment and 29 dying after conservative treatment. Among the 95 surviving patients, the mean follow-up time was 14.5 ± 1.8 months. In the surgical group, 42 patients had one-stage wound healing, two did not heal owing to subcutaneous stent provocation, which healed after removing the INFIX, and six patients with lateral femoral cutaneous nerve paralysis recovered after removal of the INFIX. The average time for bed rest and clinical fracture healing in the operation group was shorter than that of the conservative group, with statistically significant differences between the two groups (Tables 3). The VAS scores after treatment were lower in the surgical group than in the conservative group, and the differences in scores between the two groups at one and three months post-treatment were statistically significant; however, the differences in scores between the two groups at one week and six months post-treatment were not statistically significant (Tables 4). The difference in Majeed scores after treatment between the surgical and conservative groups was statistically significant. However, the emergency admission scores between the two groups did not significantly differ (Tables 5). See Figs. 1 and 2 for typical cases.

**Table 3:** Average bed rest time and clinical fracture healing of patients with brittle pelvic fracture of pelvic ring in the two groups ($\overline{x} \pm s$, month)

	Number	Clinical healing time of fractures	Average time in bed
Conservative Group	44	5.2 ± 1.3	3.1 ± 0.5
Surgical Team	51	3.7 ± 0.8	1.2 ± 0.4
*t* value		6.872	20.566
*P* value		*P* < 0.01	*P* < 0.01

**Table 4:** Comparison of VAS between two groups of patients with fragility fractures of pelvic ring at different times ($\overline{x} \pm s$, score)

	Number	1 week	1 month	3 months	6 months	*F* value	*P* value
Conservative Group	44	2. 8 ± 1. 3	2. 2 ± 0.9	1. 5 ± 0.8	0. 6 ± 0.3	48.813	< 0.001
Surgical Team	51	2. 6 ± 1. 2	1. 9 ± 0.5	1. 2 ± 0. 6	0. 5 ± 0. 4	71.000	< 0.001
*t* value		0.779	2.04	2.084	1.360		
*P* value		0.438	0.044	0.040	0.177

**Table 5:** Comparison of the Majeed functional score between two groups of patients with fragility fractures of the pelvic ring ($\overline{x} \pm s$, score)

	Number	On admission	1 week	1 month	3 months	6 months	*F* value	*P* value
Working								
Conservative Group	44	1.0 ± 0.9	2.5 ± 1.2	3.1 ± 0.8	4.3 ± 1.5	12.3 ± 5.8	112.031	** < 0.001**
Surgical Team	51	1.5 ± 1.1	2.9 ± 1.5	4.2 ± 1.2	6.2 ± 1.9	15.8 ± 4.1	325.065	** < 0.001**
*t* value		− 2.400	− 1.423	− 5.168	− 5.348	− 3.430		
*P* value		0.018	0.159	< 0.01	< 0.01	0.01		
Pain								
Conservative Group	44	8.5 ± 2.6	13.6 ± 1.9	15.3 ± 3.6	21.6 ± 3.5	26.2 ± 2.9	240.670	** < 0.001**
Surgical Team	51	7.9 ± 2.1	15.7 ± 3.2	22.9 ± 4.2	25.6 ± 2.9	27.2 ± 1.7	375.000	** < 0.001**
* t* value		1.244	− 3.949	–9.389	− 0.81	− 2.083		
* P* value		0.217	< 0.001	< 0.001	0.936	0.040		
Sitting								
Conservative Group	44	3.2 ± 0.9	7.2 ± 0.8	10.8 ± 2.4	15.3 ± 5.6	24.2 ± 1.8	344.065	** < 0.001**
Surgical Team	51	2.9 ± 1.1	13.5 ± 3.9	20.5 ± 4.5	25.8 ± 3.7	26.8 ± 2.1	457.133	** < 0.001**
* t* value		1.440	1.409	− 10.519	− 12.807	− 5.150		
* P* value		0.136	0.162	< 0.01	< 0.01	< 0.01		
Sexuality								
Conservative Group	44	0.0	0.0	0.0	0.2 ± 0.1	4.2 ± 1.3	446.729	** < 0.001**
Surgical Team	51	0.0	0.0	0.0	2.5 ± 0.8	4.5 ± 1.5	368.382	** < 0.001**
*t* value		− 6.126	− 6.126	− 6.126	− 6.424	− 2.067		
*P* value		< 0.01	< 0.01	< 0.01	< 0.01	0.042		
Total score								
Conservative Group	44	14.1 ± 3.1	25.9 ± 2.6	32.0 ± 4.5	45.7 ± 6.9	75.0 ± 5.9	1007.43	** < 0.001**
Surgical Team	51	13.9 ± 2.8	34.8 ± 5.4	51.8 ± 6.4	66.2 ± 5.3	82.5 ± 5.2	1368.04	< 0.001
* t* value		0.496	− 10.034	− 17.635	− 16.72	− 6.586		
* P* value		0.621	< 0.01	< 0.01	< 0.01	< 0.01		

Bold values indicate repeated measurement analysis of variance to compare whether there was any difference at each time point, and *P* value <0.001 suggested that there was statistical difference

## Discussion

With an ageing population, the number of geriatric pelvic fractures increases yearly. Unlike hip fractures, pelvic fractures are associated with a higher mortality risk [20]. Additionally, older patients are more prone to perioperative accidents owing to declining physical function and decreased resistance, resulting in poor survival rates and quality of life [21–23]. Rommens et al. [24] proposed the early fixation of FFP in older populations using minimally invasive techniques, with the aim of fixation and early restoration of mobility rather than anatomical repositioning. The principles are similar to those used to treat fragility fractures of the hip in the older population.

With advances in minimally invasive pelvic techniques for anterior ring injuries, Vaidya [18] first proposed the INFIX technique. This technique overcomes the disadvantages of anterior ring pin tract infection in external fixation, restriction of hip movement and difficulties in care. The INFIX has been biomechanically shown to have comparable stability to the mechanical advantages of double plates, superior to single plates and external fixation frames, and better post-operative patient comfort than external fixation frames [25]. In addition, the INFIX has many advantages over previous methods, such as less trauma, less bleeding, fewer complications and shorter operative time. Therefore, the possibility of internal fixation loosening and failure owing to osteoporosis can be reduced, and improvements in the quality of life and survival rate of older patients can be observed [26, 29].

Although additional clinical and biomechanical work is required to identify the optimal stabilisation procedure for FFP, the developed procedures demonstrate a shift from open reduction towards closed internal fixation. Lodde et al. [27] compared five fixation techniques for unstable fragile fractures of the pelvic ring and observed that a retrograde transpubic screw, or S1/S2 ala-ilium screws, can be considered viable alternatives in cases where minimally invasive techniques are applicable. Hopf et al. [28] reported that 30 patients with posterior ring fractures were treated using percutaneous iliosacral screw fixation, and intra- and post-operative complications were rare. This was in accordance with our results of pain relief after surgical intervention in all patients. Shetty et al. [29] used the INFIX and percutaneous iliosacral screws and achieved optimal fracture reduction and definitive stabilisation with minimum complications and excellent functional outcomes. Most of our patients experienced similar treatment results for post-operative complications and function scores. Biomechanical studies have demonstrated that the stability of iliosacral screw fixation is significantly higher when combined with cement augmentation [30]. In our study, we used the INFIX and the sacroiliac screw technique for anterior and posterior ring injuries without cement because cement easily leaks into the sacral canal, the neuroforamina or through the anterior sacral cortex. Compared with sacroplasty, the injected cement may hinder fracture healing and be an obstacle for iliosacral screw osteosynthesis when additional fracture stabilisation is required [31]. Therefore, further investigation is required to determine the optimal technique. Recent studies using CT fluoroscopy-guided screw placement have demonstrated promising post-operative results with a low complication rate [32]; however, higher effective doses of radiation used for intraoperative CT navigation must be considered [33].

Minimally invasive reduction and fixation is a novel technology which primarily relies on the intraoperative perspective machine. Malpositioning of S1 and S2 screws can be a devastating complication, with possible damage to the iliac and superior gluteal vessels, the L5–S1 nerve roots and the sympathetic chain [34]. In recent years, newer techniques have been developed based on computer-assisted and two-dimensional and 3D fluoroscopy-based navigation. After CT data are obtained, navigation is guided using the data to determine the surgical corridor for screw placement [35, 36]. A prospective study demonstrated that placement of percutaneous iliosacral screws determined using the navigation system improved screw positioning in dysmorphic sacra but came at the cost of a longer operative time and increased radiation exposure to the patient [37]. Authors have also proposed that the emergence of navigation technology could solve these problems. Sacroiliac screw implantation assisted by robot and 3D-printed templates can significantly shorten operation time, reduce patient radiation exposure, and improve safety and accuracy [38]. Chao et al. [39] used a patient-specific locked navigation template to treat pelvic fracture or sacroiliac dislocation with dysplastic sacrum. They demonstrated that the method was clinically safe using finite element analysis. Therefore, we can also use the new technology to reduce the learning curve of doctors, reduce the operation time on older patients, and improve the treatment effect. However, we should also consider the disadvantages of the new technologies, such as increased radiation exposure and high cost.

Aiming to stabilise and reset the fracture early, non-absolute anatomical repositioning, reduce the duration of surgical anaesthesia, reduce the risk of surgery, promote early functional exercise, reduce bed rest, prevent bed rest-related complications in the medium and long term, and improve patients’ quality of life and survival rates. A preoperative assessment should be completed considering a full range of factors, such as advanced age, fracture site, thrombosis risk assessment [40], albumin level, anaemia, heart disease, respiratory disease, history of stroke, smoking, BMI, Parkinson’s disease and osteoporosis, to prevent, control and manage risk factors, adopt a similar treatment model and thinking to that of treating elderly hip fractures and establish a fast-track system. Compared with conservative treatment, surgery substantially reduces the bed rest time, improves the quality of life of patients, restores most of their self-care ability and reduces the burden on their families and society.

Therefore, the geriatric hip fracture treatment model combined with minimally invasive pelvic repositioning and fixation can be used to treat FFP in the older population [31]. Joint participation in the diagnosis and rehabilitation of patients with pelvic fractures will be the focus of subsequent clinical work.

However, our study has limitations, such as the short follow-up period and small number of cases. Furthermore, most patients receiving conservative treatment have more comorbidities and are more severely ill, which may lead to a higher mortality rate.

## Conclusion

Early and proper fixation of the fracture and reduced bed rest time are beneficial for the quality of life and survival after the fracture. Therefore, our findings suggest that minimally invasive surgery for treating FFP in the older population may be a trend in the future.

## Data Availability

All data generated or analysed during this study are included in this published article.
